# Differential effects of apelin-13 on lipid peroxidation and DNA oxidation in doxorubicin-treated rats: A preliminary study

**DOI:** 10.17305/bb.2025.13217

**Published:** 2025-12-09

**Authors:** Katarzyna Matusik, Katarzyna Kamińska, Kaja Kasarełło, Agnieszka Cudnoch-Jędrzejewska

**Affiliations:** 1 Chair and Department of Experimental and Clinical Physiology, Laboratory of Centre for Preclinical Research, Medical University of Warsaw, Warsaw, Poland

**Keywords:** Apelin-13, doxorubicin, oxidative stress.

## Abstract

Doxorubicin-induced cardiotoxicity is closely associated with oxidative stress (OS), and apelin-13 has been proposed as a potential cardioprotective peptide. However, its effects on specific OS markers remain poorly understood. This preliminary study aimed to evaluate the impact of apelin-13 on OS markers in rats chronically treated with doxorubicin (DOX). Male rats received DOX with or without apelin-13 (40 µg/kg body weight/day). The levels of 8-hydroxy-2’-deoxyguanosine (8-OHdG) and malondialdehyde (MDA) were measured as indicators of oxidative DNA damage and lipid peroxidation, respectively. The DOX treatment resulted in increased MDA levels, which were unaffected by apelin-13. Conversely, 8-OHdG levels decreased with DOX alone but returned to baseline levels in the presence of DOX and apelin-13. In conclusion, while apelin-13 did not mitigate DOX-induced lipid oxidative damage, it may selectively influence nuclear OS markers. This suggests a complex and context-dependent role of apelin-13 in modulating OS associated with DOX treatment.

## Introduction

Doxorubicin (DOX), a member of the anthracycline family, is among the most effective anticancer agents; however, its clinical application is significantly hindered by cardiotoxicity, which is linked to increased oxidative stress (OS) in myocardial cells [1]. The toxic effects of DOX are primarily associated with the overproduction of reactive oxygen species (ROS), resulting in lipid peroxidation, DNA damage, and mitochondrial dysfunction [2]. Indicators of such damage include malondialdehyde (MDA), a marker of lipid peroxidation, and 8-hydroxy-2′-deoxyguanosine (8-OHdG), a product of oxidative DNA modification [3].

Apelin, an endogenous ligand of the G protein-coupled apelin receptor (APJ), plays a crucial role in regulating cardiovascular function and metabolism [4]. Recent studies have demonstrated that the apelin isoform, apelin-13, can exert a cardioprotective effect by mitigating DOX-induced oxidative and apoptotic damage [5, 6]. Nonetheless, the mechanisms underlying this protection, particularly regarding its effects on OS markers such as MDA and 8-OHdG, remain insufficiently understood.

This study aimed to evaluate the impact of apelin-13 on OS markers in rats administered DOX.

## Materials and methods

### Animals

The study involved 24 male Sprague-Dawley rats (12 weeks old, weighing 250–300 g). All procedures were approved by the II Local Animal Ethics Committee (No. WAW2/087/2021, dated 02.06.2021). The animals were bred and maintained at the Central Laboratory of Experimental Animals (Medical University of Warsaw) under standard conditions (22–24 ^∘^C, 12-h light/dark cycle, 45%–65% humidity) with ad libitum access to food and water.

### Experimental design

The animals were randomly assigned to three groups (*n* ═ 8). Over 28 days, the animals received a continuous infusion of either saline (NaCl, DOX groups) or apelin (APE40 group). Apelin was administered as Apelin-13 trifluoroacetate (TFA) salt (Cat. No.: HY-P1944A, MedChemExpress, Sollentuna, Sweden) at a dose of 40 µg/kg body weight/day. Saline and apelin were delivered via osmotic pumps (Alzet Corp., Cupertino, CA, USA; model 2ML4), which were implanted subcutaneously on the back. All animals received intraperitoneal injections once a week on days 1, 8, 15, and 22. The NaCl group received saline injections, while the DOX and APE40 groups received DOX at a dose of 3.5 mg/kg body weight per week (Cat. No.: HY-15142, MedChemExpress, Sollentuna, Sweden).

### Enzyme-linked immunosorbent assay (ELISA)

The levels of MDA and 8-OHdG in left ventricular (LV) homogenates were analyzed using commercial kits, following the manufacturer’s instructions (Double Antibody Sandwich Rat MDA ELISA Kit, ca. no. EIA06027r; Wuhan Newqidi Biotech Co., Ltd., Wuhan, China; Double Antibody Sandwich Rat 8-OHdG ELISA Kit, ca. no. EIA05009r; Wuhan Newqidi Biotech Co., Ltd., Wuhan, China).

### Compliance with ethical standards

All experimental procedures were approved by the II Local Animal Research Ethics Committee (Application No. WAW2/087/2021, release date: 02.06.2021).

### Statistical analysis

Data sets passed the normality test (Shapiro-Wilk test), and thus one-way analysis of variance (ANOVA) with Sidak’s post hoc test was employed for statistical analysis. Values in the figures are presented as medians with interquartile ranges (IQRs).

## Results

**Figure 1.**
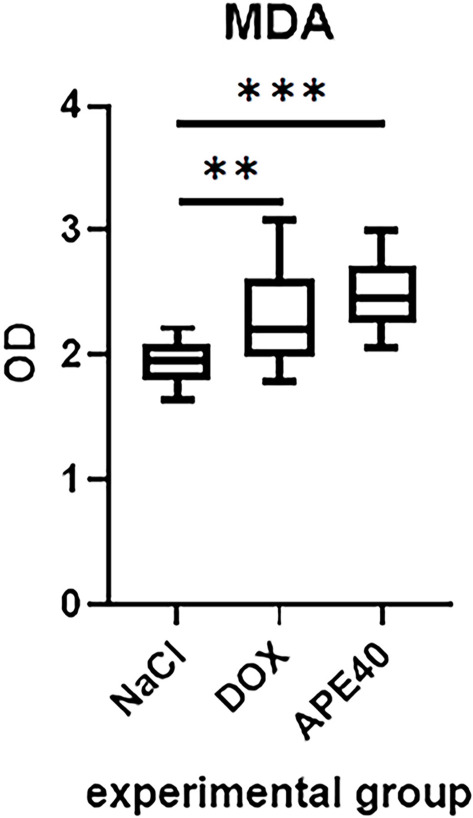
**Levels of malondialdehyde (MDA) in the left ventricle (LV) of rats from the NaCl group, the doxorubicin-treated (DOX) group, and the doxorubicin- and apelin-13-treated (APE40) group, presented as optical density (OD) values.** Data are presented as medians with interquartile ranges (IQR), derived from two technical replicates per animal. The number of technical replicates included in the analysis was as follows: NaCl: *n* ═ 14, DOX: *n* ═ 16, APE40: *n* ═ 15.

DOX treatment significantly increased MDA levels compared to the NaCl group (**P═0.0064). The APE40 group also exhibited significantly elevated MDA levels compared to the NaCl group (***P<0.0001). However, no statistically significant difference was observed between the DOX and APE40 groups (*P*═0.2267) (Figure 1).

**Figure 2.**
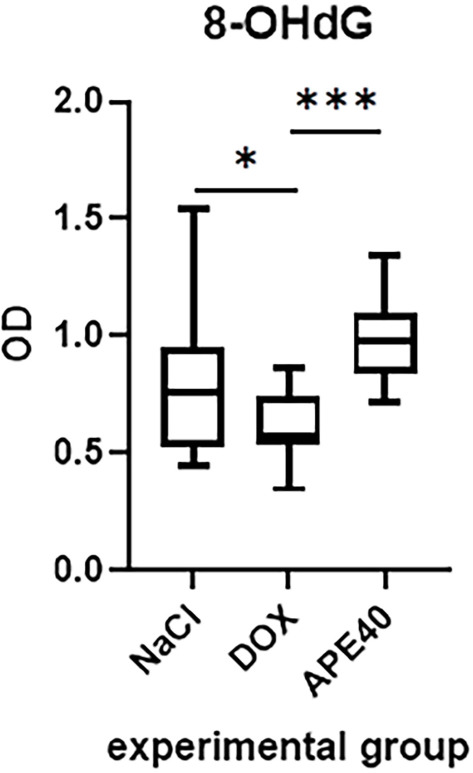
**Levels of 8-hydroxy-2’-deoxyguanosine (8-OHdG) in the left ventricle (LV) of rats from the NaCl group, the doxorubicin-treated (DOX), and the doxorubicin- and apelin-13-treated (APE40) group, presented as optical density (OD) values.** Data are presented as medians with interquartile ranges (IQR), derived from two technical replicates per animal. The number of technical replicates included in the analysis was as follows: NaCl: *n* ═15, DOX: *n* ═ 15, APE40: *n* ═ 16.

The DOX group showed a significant reduction in 8-OHdG levels compared to the NaCl group (*P═0.0445). The administration of apelin-13 (APE40) restored 8-OHdG levels to values comparable to those observed in the NaCl group (*P*═0.1797). Moreover, 8-OHdG levels in the APE40 group were significantly higher than those in the DOX group (***P═0.0002) (Figure 2).

## Discussion

Previous studies have demonstrated that apelin-13 exerts protective effects in cardiac models by reducing ROS, MDA and inducing the activity of antioxidant enzymes, including superoxide dismutase, catalase, and glutathione peroxidase. Additionally, apelin-13 activates phosphoinositide 3-kinase/protein kinase B (PI3K/Akt) signaling and delays the opening of the mitochondrial permeability transition pore (MPTP) [7]. However, in our experiment, apelin-13 did not reduce MDA levels (Figure 1), suggesting that the oxidative mechanisms induced by DOX may be resistant to its effects. Nonetheless, the modulation of 8-OHdG levels (Figure 2) indicates that apelin-13 may influence nuclear or mitochondrial responses, thereby affecting markers of DNA damage despite the absence of an anti-lipid effect.

Our data suggest that the role of apelin-13 in oxidative protection against DOX is more intricate than previously anticipated and may depend on the type of OS and the specific tissue or damage type. Similar findings were reported by Duan et al. (2019), who demonstrated that apelin-13 reduced MDA levels and oxidative damage in a model of ischemic stroke [8], and by Xu and Li (2020), who observed decreased OS markers following apelin-13 treatment in spinal cord ischemia-reperfusion injury [9]. In contrast, our results imply that apelin-13 may exert more selective, context-dependent effects on nuclear rather than lipid oxidative damage. Possible explanations for these discrepancies include differences in subcellular targets of apelin-13, with greater efficacy on nuclear compartments compared to membrane lipids. Apelin-13 may enhance DNA repair pathways or preferentially modulate nuclear ROS levels without significantly impacting mitochondrial lipid peroxidation, as reflected by MDA levels. These findings may have translational relevance for patients undergoing long-term DOX therapy. While apelin-13 did not attenuate lipid peroxidation, its ability to restore 8-OHdG levels suggests potential modulatory effects on nuclear OS and DNA integrity. Understanding these mechanisms could inform the development of adjunctive strategies aimed at minimizing DOX-induced cellular damage without compromising its antitumor efficacy.

## Conclusion

This preliminary study aimed to investigate the potential role of apelin-13 during chronic DOX treatment. Although apelin-13 did not mitigate DOX-induced lipid oxidative damage, it may selectively influence nuclear OS markers. This suggests a complex and context-dependent role for apelin-13 in modulating OS associated with DOX treatment. However, the limitations of using a single dose, measuring only two biomarkers, and the absence of functional or histopathological assessments weaken the conclusions drawn. Further studies are necessary to confirm these findings and elucidate the underlying mechanisms involved.

## Data Availability

The corresponding author, Katarzyna Kamińska, will provide the data that supports the findings of this study upon request.
